# Physiological stability and renal clearance of ultrasmall zwitterionic gold nanoparticles: Ligand length matters

**DOI:** 10.1063/1.4978381

**Published:** 2017-05-01

**Authors:** Xuhui Ning, Chuanqi Peng, Eric S. Li, Jing Xu, Rodrigo D. Vinluan, Mengxiao Yu, Jie Zheng

**Affiliations:** Department of Chemistry and Biochemistry, The University of Texas at Dallas, 800 W Campbell Rd., Richardson, Texas 75080, USA

## Abstract

Efficient renal clearance has been observed from ultrasmall zwitterionic glutathione-coated gold nanoparticles (GS-AuNPs), which have broad preclinical applications in cancer diagnosis and kidney functional imaging. However, origin of such efficient renal clearance is still not clear. Herein, we conducted head-to-head comparison on physiological stability and renal clearance of two zwitterionic luminescent AuNPs coated with cysteine and glycine-cysteine (Cys-AuNPs and Gly-Cys-AuNPs), respectively. While both of them exhibited similar surface charges and the same core sizes, additional glycine slightly increased the hydrodynamic diameter of the AuNPs by 0.4 nm but significantly enhanced physiological stability of the AuNPs as well as altered their clearance pathways. These studies indicate that the ligand length, in addition to surface charges and size, also plays a key role in the physiological stability and renal clearance of ultrasmall zwitterionic inorganic NPs.

Ultrasmall zwitterionic luminescent metal nanoparticles (NPs) are highly resistant to serum protein binding, efficiently renal clearable with low systemic accumulation.^1–3^ Glutathione (GSH) is a commonly used zwitterionic ligand for stabilizing inorganic metal nanoparticles in the physiological environment due to its abundance in the body and antifouling properties.^4^ For example, we were able to synthesize GSH coated gold nanoparticles (GS-AuNPs), sliver nanoparticles (GS-AgNPs), and copper nanoparticles (GS-CuNPs) of 1.7∼3 nm, which exhibited strong resistance to serum protein binding and effective renal clearance of >50% ID (injection dose) at 24 h post-injection (p.i.).^5–7^ Similar renal clearance was observed from even smaller GS-AuNPs.^8^ Efficient renal clearance of these zwitterionic metal NPs also found exciting applications in cancer diagnosis and kidney functional imaging. For example, by utilizing the enhance permeability and retention (EPR) effect, GS-AuNPs could passively target tumors with 10 times higher concentration over IRDye 800CW at 12 h p.i. and were able to be cleared from normal tissue >3× faster within 24 h p.i. due to their effective renal clearance ability. By taking advantage of highly sensitive fluorescence kidney function imaging, GS-AuNPs can noninvasively detect the different stages of kidney dysfunction by comparing fluorescence peak value, relative renal function (RRF), clearance percentage, and peak time.^9,10^

While these zwitterionic metal NPs hold great promise in the future clinical translation, the fundamental understanding on the origin of efficient renal clearance is still not clear. In the past decade, size and surface charge effect have been mainly attributed to their low serum protein adsorption and highly efficient renal clearance. For example, by comparing the GS-AuNPs with hydrodynamic diameters (HD) of 6 and 13 nm, we found that accumulation of 6 nm GS-AuNPs in the urine and liver was 4.0% and 27.1%, while 0.5% and 40.5% of 13 nm GS-AuNPs were in urine and liver, respectively, indicating that nanoparticles of smaller sizes favored renal clearance.^7^ By coating quantum dots with charged molecules (dihydrolipoic acid as anionic, cysteamine as cationic, cysteine as zwitterionic, and polyethylene glycol as neutral), Choi *et al.* found that charge of coating had a dramatic effect on serum protein binding. Unlike purely anionic or cationic charges that increase the binding affinity of quantum dots with serum protein, zwitterionic surface coating prevents serum protein adsorption while maintaining high solubility in the physiological environment.^11^ While these studies have greatly advanced our fundamental understanding on renal clearance of ultrasmall NPs, it is not clear whether the ligand length will have impact on the physiological stability and renal clearance. To address this question, in this work, we prepared same sized and charged zwitterionic luminescent gold nanoparticles coated with cysteine and glycine-cysteine (Cys-AuNPs and Gly-Cys-AuNPs), respectively. Surprisingly, the single amino-acid increase in length from cysteine (0.47 nm) to glycine-cysteine (0.85 nm) induced significantly enhanced stability in physiological conditions for Gly-Cys-AuNPs over Cys-AuNPs even though the hydrodynamic diameter (HD) of AuNPs was only increased about 0.4 nm (Figs. 1(a) and 1(d)). Although pharmacokinetics study showed similar distribution and elimination half-lives, the biodistribution study unveiled that hepatic uptake of AuNPs was significantly minimized and renal clearance was greatly enhanced once surface ligands of AuNPs were changed from Cys to Gly-Cys.

The luminescent Cys-AuNPs and Gly-Cys-AuNPs were synthesized by mixing the ligand (Cys or Gly-Cys) aqueous solution (180 μl, 25 mM) with HAuCl_4_ (200 μl, 25 mM), and polymeric Cys-Au(I) and Gly-Cys-Au(I) were formed immediately at room temperature with a color of bright orange, which eventually formed Cys-AuNPs and Gly-Cys-AuNPs. The final solution was left to react at room temperature for 3 days. The solution was then centrifuged to remove large NPs and polymers. The supernatant was then purified by the size exclusion column to remove all free gold ions and ligands.

The ultrasmall luminescent Cys-AuNPs and Gly-Cys-AuNPs exhibited identical core sizes from transmission electron microscopy (TEM) measurement, which were 2.35 ± 0.33 nm and 2.32 ± 0.19 nm, respectively (Figs. 1(b), 1(c), 1(e), and 1(f)). Meanwhile, the Cys-AuNPs showed the HD of 2.69 ± 0.46 nm in an aqueous solution from dynamic light scattering analysis (DLS, Brookhaven 90Plus Dynamic Light Scattering Particle Size Analyzer), whereas the HD of Gly-Cys-AuNPs was 3.12 ± 0.61 nm (Figs. 1(c) and 1(f)). The increase of ∼0.4 nm in HD for Gly-Cys-AuNPs comparing with Cys-AuNPs was mainly due to the increase of surface ligand length (by one amino-acid). This was consistent with the previous studies on HDs of quantum dots coated with different lengths of capping ligands (TOPO, DHLA, DHLA-PEG600, DHLA-PEG1000, MUA, and DDPE-PEG2000), where a systematic increase in hydrodynamic radius was also observed.^12^

**FIG. 1.**
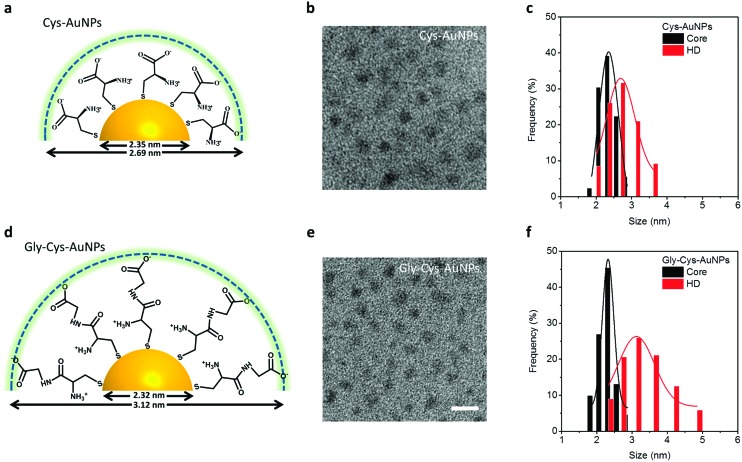
Scheme, TEM image, and size analysis of ((a)-(c)) Cys-AuNPs and ((d)-(f)) Gly-Cys-AuNPs (scale bar for TEM images in (b) and (e): 5 nm).

Both Cys-AuNPs and Gly-Cys-AuNPs exhibited a red colored emission with a maximum of 650 nm (Figs. 2(a) and 2(b)); however, the Cys-AuNPs had a lower quantum efficiency of 0.24% compared to that of Gly-Cys-AuNPs of 0.42%. Both particles showed the maximum excitation localized at 450 nm and a Stokes shift of 200 nm, indicating the metal-to-ligand charge transfer (Au to S). The high similarity observed in excitation and emission of Cys-AuNPs and Gly-Cys-AuNPs suggested that optical transitions among AuNPs with these two different surface coatings were mainly governed by Au-S charge transfer.^13,14^

**FIG. 2.**
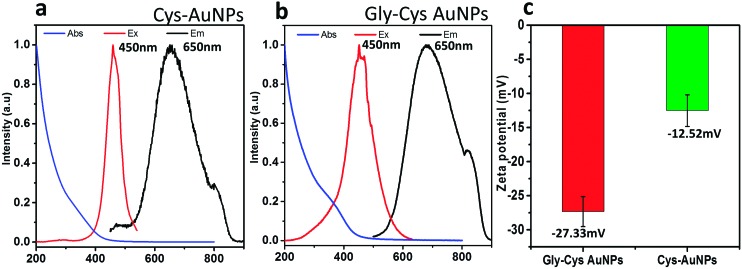
Absorbance, excitation, and emission spectra of (a) Cys-AuNPs and (b) Gly-Cys-AuNPs. Excitation spectra: emission at 650 nm; emission spectra: excitation at 450 nm. (c) Zeta potential measurement of Cys-AuNPs and Gly-Cys-AuNPs at pH 9, where the maximum stability of Cys-AuNPs was achieved.

To understand how the surface ligand length affected the renal clearance and bio-distribution, we first investigated the stability of the different ligand-coated particles in different pH. As seen in Fig. 3(a), Cys-AuNPs were highly unstable and tended to aggregate once the pH was below 8.5, and the aggregation process is reversible as shown in Fig. 3(b) where the same batch of Cys-AuNPs was adjusted repeatedly from pH 9 to 7 and HD of the Cys-AuNPs remains to be 2.6 ± 0.35 nm at pH 9 and 225 ± 30 nm at pH 7. Gly-Cys-AuNPs however maintained the constant HD of ∼3 nm in different pH. The desired stability of Gly-Cys-AuNPs compared with Cys-AuNPs was mainly attributed to the increase in hydrodynamic diameter of 0.4 nm, even though both of them were zwitterionic in pH 7.4. In order to compare the surface charge of the two different particles, the zeta potential was measured at pH 9, where the stability of Cys-AuNPs was optimized; Gly-Cys-AuNPs held a much negative zeta potential of −27.33 mV, whereas that of Cys-AuNPs was −12.52 mV as shown in Fig. 2(c). This could be explained by the different pKa values of the amine groups: the pKa of amine on Gly-Cys-AuNPs and Cys-AuNPs is about 9.78 and 10.25, respectively; therefore, there are more positively charged amines on Cys-AuNPs than Gly-Cys-AuNPs in pH 9, which results in the difference in the net surface charge.

**FIG. 3.**
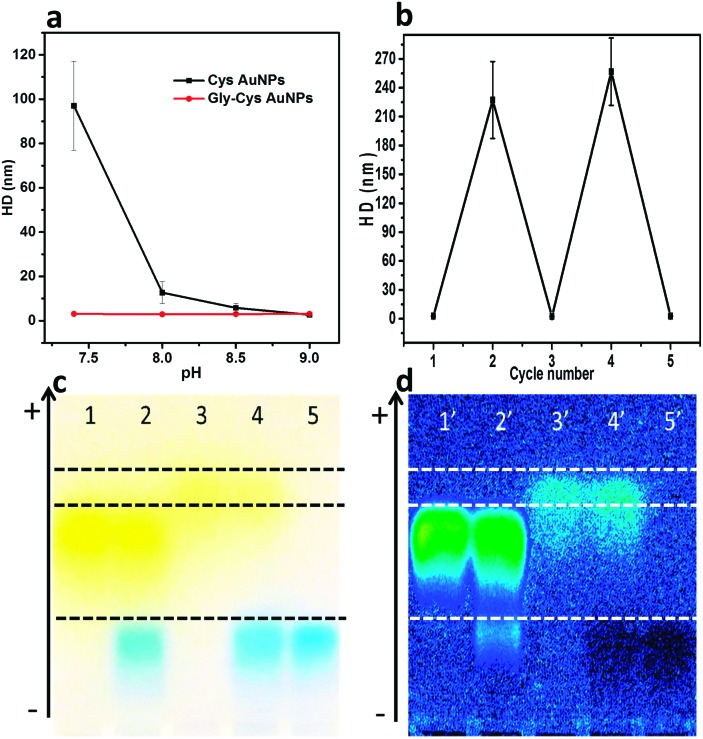
(a) HDs of Cys-AuNPs and Gly-Cys-AuNPs in different pH measured by DLS. (b) HD of Cys-AuNPs measured between pH 9 and 7 for reversibility. ((c) and (d)) Optical and fluorescence images of gel electrophoresis: 1 and 1’, Cys-AuNPs; 2 and 2’, Cys-AuNPs with 10% FBS and CBB; 3 and 3’, Gly-Cys-AuNPs; 4 and 4’, Gly-Cys-AuNPs with 10% FBS and CBB; 5 and 5’, FBS and CBB. For fluorescence imaging, excitation at 420 nm; emission at 600 nm.

To further investigate the serum protein interaction with Cys-AuNPs and Gly-Cys-AuNPs, gel electrophoresis was conducted further (Figs. 3(c) and 3(d)). Firstly, Cys-AuNPs (band 2) and Gly-Cys-AuNPs (band 4) were incubated with 10% fetal bovine serum (FBS, v/v) at 37 °C for 30 min; free Cys-AuNPs (band 1), Gly-Cys-AuNPs (band 3), and FBS (band 5) in PBS were used as control, and Coomassie Brilliant Blue (CBB) dye was added to stain FBS (in band 2, 4, 5). The overall pH of the gel and buffer was adjusted to 8 to prevent aggregation of Cys-AuNPs to minimize interference to the serum protein study. The optical image of gel electrophoresis showed that both AuNPs had minimized serum protein interaction. However, with fluorescence imaging, we identified that very small fraction of Cys-AuNPs still bounded to serum protein. While no serum protein adsorption was observed for Gly-Cys-AuNPs. These results indicated that the increased ligand length from Cys to Gly-Cys not only could stabilize the particles at physiological pH but also further improve the serum protein resistance.

During the *in vivo* study with murine model, distinct clearance pathways were observed for these two AuNPs. The female balb/c mice were administrated with Cys-AuNPs or Gly-Cys-AuNPs intravenously, and urine and blood samples were collected at different time points within 24 h p.i. (n = 3). For biodistribution study, the mice were euthanized at 24 h p.i. and organs were collected. The urine, blood, and organ samples were dissolved in aqua regia, and gold concentrations were analyzed by inductively coupled plasma mass spectrometry (ICP-MS). As shown in Fig. 4(a), both of AuNPs with the same size and surface charges exhibited rapid elimination and short retention in blood with two compartment pharmacokinetics, and their distribution half-lives(${\text{t}}_{1/2\alpha }$) were 3.18 min and 2.46 min for Cys-AuNPs and Gly-Cys-AuNPs, respectively, indicating a fast distribution of particles in the body. In addition, the elimination half-lives $\left({\text{t}}_{1/2\beta }\right)$ of Cys-AuNPs (4.93 h) were comparable to that of Gly-Cys-AuNPs (4.25 h). In order to gain more quantitative understanding of their *in vivo* behaviors, we compared the renal clearance and biodistribution of these two AuNPs. The Gly-Cys-AuNPs showed efficient renal clearance with an efficiency of 41.6% ID at 24 h p.i, which was 1.93× higher than Cys-AuNPs with a renal clearance efficiency of 21.5% (Fig. 4(b)). The low renal clearance of Cys-AuNPs was consistent with their biodistribution. The accumulation of Cys-AuNPs in liver and spleen was 28.1% and 14.2% ID/g, which were 23.6 and 4.1× higher than those of Gly-Cys-AuNPs (1.19% ID/g in liver and 3.44% ID/g in spleen), respectively (Fig. 4(c)). Since the liver is a key organ in the hepatic clearance route as well as kidneys for the renal elimination, by further investigating the organ-to-blood ratio in Fig. 4(d), we found that a liver-to-blood ratio of Cys-AuNPs was 18.7, which was 22× higher than that of Gly-Cys-AuNPs (0.85). This further indicated that Cys-AuNPs held much higher affinity to the liver uptake than Gly-Cys-AuNPs. In addition, the kidney-to-blood ratio of Cys-AuNPs (10.1) was 1.9× higher than that of Gly-Cys-AuNPs (5.3), which indicated that the Cys-AuNPs also have longer retention in the kidney than Gly-Cys-AuNPs.

**FIG. 4.**
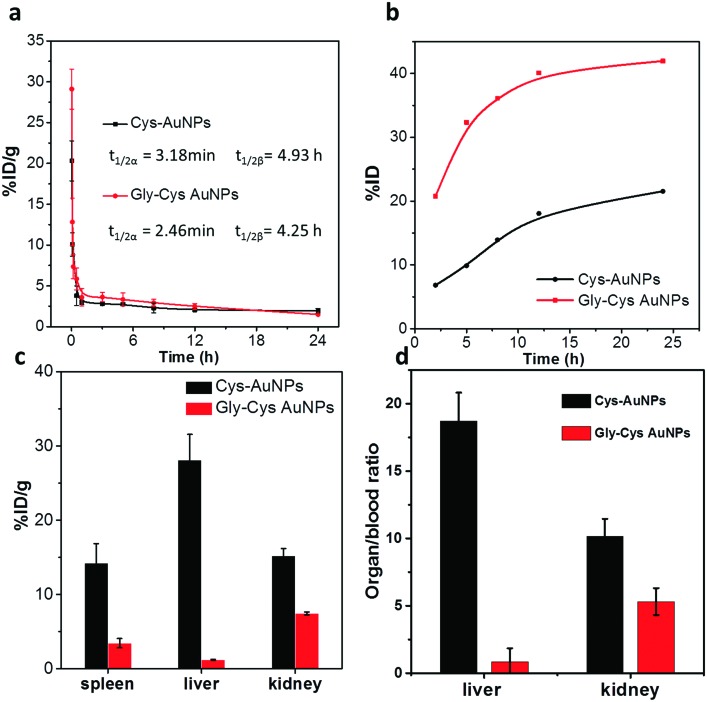
((a) and (b)) Pharmacokinetics and renal clearable of Cys-AuNPs and Gly-Cys-AuNPs in balb/c mice within 24 h p.i. (n = 3). (c) Distribution of Cys-AuNPs and Gly-Cys-AuNPs in liver, spleen, and kidney at 24 h p.i. (n = 3). (d) Liver-to-blood and kidney-to-blood ratios of Cys-AuNPs and Gly-Cys-AuNPs at 24 h p.i.

In conclusion, to understand the origin of high renal clearance efficiency observed from ultrasmall zwitterionic glutathione-coated AuNPs, we synthesized Cys-AuNPs and Gly-Cys-AuNPs with the same core size and surface charges and conducted head-to-head comparison on their physiological stability, renal clearance, pharmacokinetics, and biodistribution. Our results show that introducing an additional amino acid, glycine, can significantly enhance the stability of zwitterionic AuNPs in physiological pH and improve their resistance to serum protein adsorption. More importantly, this additional glycine also minimizes the hepatic uptake of AuNPs and significantly enhances the renal clearance of zwitterionic AuNPs. This fundamental understanding of how the ligand length affects the behavior of nanoparticles will lay down a foundation to future design of nanomedicine with enhanced physiological stability and renal clearance, expediting their clinical translation.
